# The Crosstalk between Mesenchymal Stem Cells and Macrophages in Bone Regeneration: A Systematic Review

**DOI:** 10.1155/2021/8835156

**Published:** 2021-06-14

**Authors:** Rita Lih-Ying Shin, Chien-Wei Lee, Oscar Yuan-Jie Shen, Hongtao Xu, Oscar Kuang-Sheng Lee

**Affiliations:** ^1^Department of Orthopaedics and Traumatology, Faculty of Medicine, Prince of Wales Hospital, The Chinese University of Hong Kong, Hong Kong SAR 999077, China; ^2^Institute for Tissue Engineering and Regenerative Medicine, The Chinese University of Hong Kong, Hong Kong SAR 999077, China; ^3^School of Biomedical Sciences, Faculty of Medicine, The Chinese University of Hong Kong, Hong Kong SAR 999077, China; ^4^Faculty of Medicine, The Chinese University of Hong Kong, Hong Kong SAR 999077, China; ^5^Li Ka Shing Institute of Health Sciences, Prince of Wales Hospital, The Chinese University of Hong Kong, Shatin, Hong Kong SAR 999077, China; ^6^Department of Orthopedics, China Medical University Hospital, Taichung, Taiwan

## Abstract

Bone regeneration is a complex and well-coordinated process that involves crosstalk between immune cells and resident cells in the injury site. Transplantation of mesenchymal stem cells (MSCs) is a promising strategy to enhance bone regeneration. Growing evidence suggests that macrophages have a significant impact on osteogenesis during bone regeneration. However, the precise mechanisms by which macrophage subtypes influence bone regeneration and how MSCs communicate with macrophages have not yet been fully elucidated. In this systematic literature review, we gathered evidence regarding the crosstalk between MSCs and macrophages during bone regeneration. According to the PRISMA protocol, we extracted literature from PubMed and Embase databases by using “mesenchymal stem cells” and “macrophages” and “bone regeneration” as keywords. Thirty-three studies were selected for this review. MSCs isolated from both bone marrow and adipose tissue and both primary macrophages and macrophage cell lines were used in the selected studies. In conclusion, anti-inflammatory macrophages (M2) have significantly more potential to strengthen bone regeneration compared with naïve (M0) and classically activated macrophages (M1). Transplantation of MSCs induced M1-to-M2 transition and transformed the skeletal microenvironment to facilitate bone regeneration in bone fracture and bone defect models. This review highlights the complexity between MSCs and macrophages, providing more insight into the polarized macrophage behavior in this evolving field of osteoimmunology. The results may serve as a useful reference for definite success in MSC-based therapy based on the critical interaction with macrophages.

## 1. Introduction

### 1.1. Fracture Healing and Bone Regeneration

Currently, over 20 million people suffer from fractures annually, predominantly due to the prevalence of osteoporosis, osteosarcoma, osteomalacia, osteomyelitis, and atrophic nonunion. Only one-quarter of these patients have received orthopedic interventions, of which more than half were treatments like bone grafting, which target the afflicted sites [1, 2]. However, the high recurrence imposes a severe economic burden on the healthcare system. To address this health problem, numerous researchers have investigated the bone regeneration process and intervention in hopes of finding more effective ways to treat these injuries.

Fracture healing is a complex and well-orchestrated process to develop the bone matrix in defective sites without forming fibrous scars, involving a series of extracellular and intracellular signaling pathways. Fracture healing can be characterized as two types: primary bone repair (direct) and secondary bone repair (indirect) [3]. Primary fracture repair does not typically occur naturally as it only occurs with rigid fixation of bone ends, direct contact, and absolute stability. On the other hand, secondary fracture repair, consisting of endochondral and intramembranous ossification, is the most common process of fracture healing and can be enhanced by load bearing and micromotion. Acute inflammatory responses within the fracture site are necessary to initiate tissue regeneration, accompanied by the secretion of proinflammatory molecules during secondary fracture repair. Biological events such as the recruitment of inflammatory cells and the promotion of angiogenesis occur after the secretion of those proinflammatory molecules. Endogenous MSCs, recruited from local soft tissues and bone marrow, migrate toward the injury site, proliferate, and differentiate into osteogenic cells. Cartilaginous callus formation provides the stable structure of the fracture site which will be replaced by a hard bony callus with more mechanical rigidity via mineralization and resorption of the soft callus. Revascularization and neoangiogenesis are also essential for fully restoring the biomechanical properties of bone [4].

### 1.2. Osteoimmunology in Bone Healing: The Role of Macrophages in Bone Healing

The entire process of fracture healing can be roughly divided into two stages: the early inflammatory phase and the tissue regeneration phase. In secondary bone repair, immune cells infiltrate the hematoma and release cytokines to initiate inflammation that is accompanied by short-lived but extensive effects on endogenous MSC recruitment and subsequent regenerative processing. Although various types of immune cells are involved [5, 6], macrophages exhibit inseparable cooperation with osteolineage cells during the whole spectrum of the fracture healing process.

Macrophage ablation reduces bone mineral density and decreased trabecular numbers during the early stage of skeletal development [7]. Schlundt et al. [8] also revealed the role of macrophages in both endochondral ossification and intramembranous ossification. Disturbed endochondral ossification due to defective cartilage resorption was observed in mice with selective macrophage depletion; meanwhile, enhanced periosteal bone formation was observed in the region distant from the fracture gap. The necessity of macrophages in both initiation and progression of early endochondral ossification was evident in a macrophage Fas-induced apoptosis (MAFIA) model [9].

Although macrophages are identified as one of the first infiltrating cells during fractures with a proinflammatory status, they also significantly regulate subsequent bone repair. Different subtypes of macrophages correspond to the stage of fracture healing. In the inflammatory phase, classically activated M1 macrophages, hereafter M1, perform phagocytosis and produce proinflammatory cytokines, such as TNF, IL-1 beta, IL-6, and IL-12, to promote osteogenesis in early and middle stages without enhancing matrix mineralization [10, 11]. In the late stage, alternatively activated macrophages, hereafter M2, release proregenerative cytokines, such as IL-10, TGF-beta, BMP2, and VEGF, to build up an anti-inflammatory environment and facilitate osteochondral differentiation and angiogenesis [5, 10]. Since both subtypes of macrophages make substantive contributions in different stages of fracture healing, regulating the presence of different macrophage subtypes is considered a therapeutic approach for fracture healing.

### 1.3. Crosstalk of Mesenchymal Stem Cells and Macrophages in Bone Healing

MSCs are regarded as a promising bioagent for treating various diseases based on their immunoregulatory capacity [12, 13]. Interestingly, the presence of macrophages is involved in the therapeutic effects of MSCs. The communication between MSCs and macrophages has been extensively studied [14]; the secretome of MSCs is altered in response to inflammatory macrophages, while a corresponding reaction of macrophages following MSC therapy is also observed—forming a feedback loop. With the emphasis on fracture healing and bone regeneration, the interaction of macrophages and MSCs has been recently summarized by Pajarinen et al., showing paracrine molecules derived from macrophages play critical roles in guiding MSC differentiation [11]. A number of reviews and systematic reviews have emphasized the role of MSCs [15–18] and macrophage polarization [19–21] in bone regeneration. However, the comprehensive understanding of the communication between MSCs and macrophages during bone regeneration remains insufficient. This review is aimed at thoroughly and systematically analyzing the communication between MSCs and macrophages in order to fill the knowledge gap of this unclarified phenomenon during bone regeneration.

## 2. Methods

### 2.1. Search Strategy

A systematic review was conducted to systematically assess articles on the crosstalk between MSCs and macrophages in bone regeneration. PubMed and Embase databases were comprehensively used to search for relevant literature by two investigators (LY Shin, HT Xu). The search term keywords are “mesenchymal stem cells” AND “macrophages” AND “bone regeneration,” combing with the mesh terms of these keywords. The details of the entire search terms and the searching workflow by PRISMA can be referred to Appendixes A–C.

### 2.2. Inclusion and Exclusion Criteria

Eligibility screening of titles and abstracts was conducted based on the following criteria: (1) articles are in English and were published in the last 10 years; (2) primary studies must be related to “mesenchymal stem cells” and “macrophages” and “bone regeneration”; and (3) review articles, case reports, letters, editorials, and correspondences were all excluded.

### 2.3. Data Extraction and Management

A standard process for data extraction of each eligible article was performed. Titles not relevant to the topic were removed first, followed by the exclusion of studies with irrelevant abstracts. All duplicates were removed. The following information was summarized from the selected studies: (1) authors, (2) cell source, (3) study type, (4) cell management, (5) interaction between MSCs and macrophages, and (6) proposed mechanisms. If there was any uncertainty or inconsistency between the reviewers (LY Shin, HT Xu), a third reviewer was consulted (CW Lee) with final identification.

### 2.4. Quality Assessment

The quality of selected papers was evaluated with a quality system constructed by Wells and Littell [22] (Appendix D). The following 8 questions were adopted in the quality scoring system. Was the study hypothesis/aim/objective clearly described? Were the experimental designs for the study well described? Were the method and materials well described? Were the time points of data collection clearly defined? Were the main outcome measurements clearly defined? Were the experimental groups well compared with the control group? Were the results well described? Was the limitation of the article discussed? Regarding each question, 1 point was allocated for “yes” and 0 points were allocated for “no.” A sum of the scores for each study was calculated independently, with a total score out of 8. Quality assessment was graded by the scores. Six to 8 was considered excellent, 4 to 6 was considered good, 2 to 4 was considered poor, and 0 to 2 was considered bad. Detailed score evaluation of selected studies can be referred to Appendix E.

## 3. Results

### 3.1. Search Results and Characteristics

**Figure 1:**
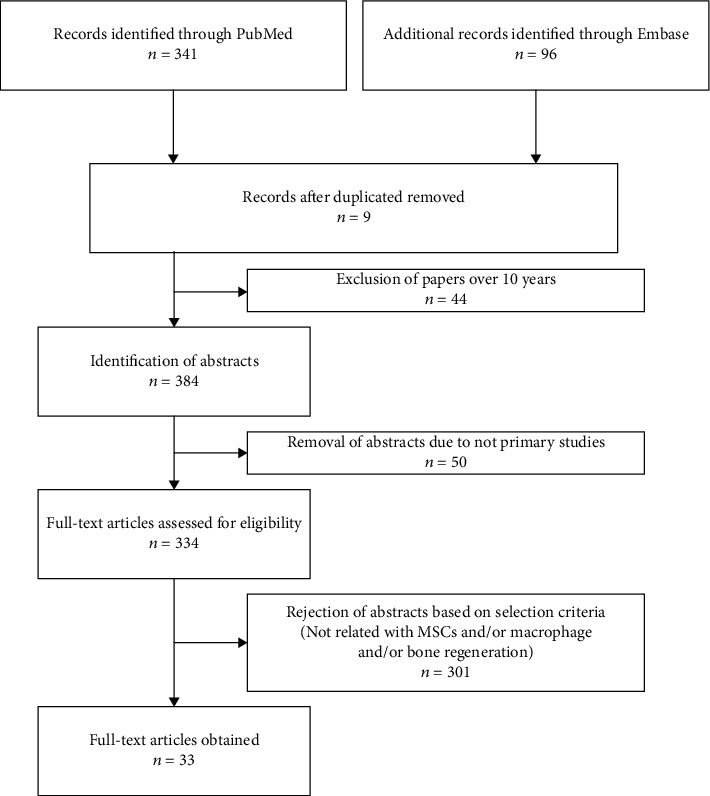
Flow diagram of the systematic review on the crosstalk of MSCs and macrophages. A total of 437 studies were retrieved based on the search strategy mentioned in the methods. Nine records after duplicates were removed. 44 works of literature published more than 10 years were excluded. After reviewing the titles and abstracts, 50 records were removed because the studies were not primary studies. After reviewing the titles and abstracts, 301 records were removed because the studies did not match the selection criteria. Finally, 33 studies met the inclusion criteria and were selected for this systematic review.

437 articles were identified in the primary searches. Two reviewers independently assessed the articles according to the inclusion and exclusion criteria to minimize bias and advance the strength of the selected articles. A joint discussion was conducted by a third reviewer when differences emerged during the assessment. After full articles were retrieved, a total of 33 studies were selected for data extraction in this review. Details of the selecting process are shown in Figure 1.

**Figure 2:**
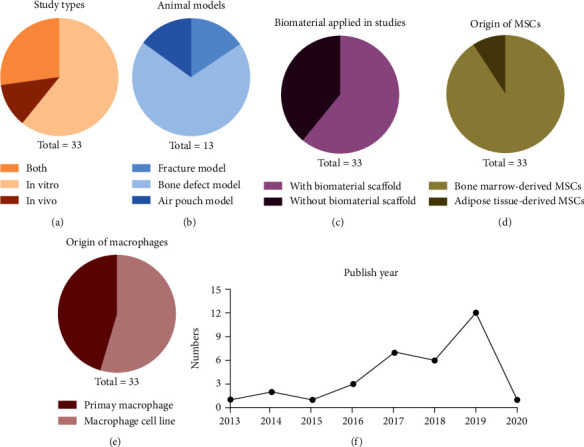
Study characteristics of the systematic review. (a) Categories of experiments. (b) Animal models of the in vivo studies. (c) The origin of the MSCs applied in studies. (d) The origin of macrophages applied in studies. (e) The proportion of biomaterials used in studies. (f) Published year of selected studies. Database searching and study identification in this review are till Jan of 2020.

All studies were published between 2013 and 2020. The categories of experiments present that 20 articles were in vitro studies, 4 articles were in vivo studies, and 9 articles applied both the in vitro and in vivo assessments. 20 articles applied biomaterial scaffolds and MSCs for bone regeneration. Among the 13 animal studies, 9 studies were using the bone defect model, 2 studies were using the fracture model, and 2 studies were using the air pouch model. MSCs derived from bone marrow were applied in whole articles, except one article that used the adipose-derived MSCs. Macrophages used in experiments can be divided into two major categories: (1) primary macrophages derived from humans or animals (mouse, rat, and rabbit) and (2) macrophage cell lines (RAW 264.7 and THP-1). Study characteristics mentioned above are summarized in Figure 2. We classified these articles into two subgroups: (1) the immunoregulatory potential of MSCs on macrophages in bone regeneration and (2) the effects of macrophages on MSC osteogenesis. Supplemental details of the experiments can be referred to Appendix F.

### 3.2. Immunoregulatory Potential of MSCs on Macrophages in Bone Regeneration

The immunomodulatory capability of MSCs and relevant effects on macrophage polarization are further discussed within this section, accompanied by the follow-up performance in bone regeneration in both the in vivo and in vitro models. Detailed results are listed in Table 1.

To uncover the subtypes of macrophages affected by exogenous MSCs, Seebach et al., Tasso et al., and Tour et al. implanted MSCs using fibrin carriers or hydroxyapatite scaffolds into bone defects. M1 macrophages and endothelial progenitor cells served as primary invaders of the bone defect site after MSC implantation in the first 2 weeks, while only a few M2 macrophages existed in the cell infiltrated area [23, 24]. M1-to-M2 macrophage switching induced by implanted MSCs has been observed in late-stage bone healing, which demonstrates that M2 macrophages prefer to accumulate in the front of cell-dense migration sites and have a proresolving phenotype that recruits vasculogenic and osteogenic progenitors from bone marrow. This M2 polarization was attributed to exogenous MSC-secreted PGE2 activating the NF-*κ*B pathway [25]. M1-to-M2 transitions are not only sequential but also closely associated with the healing process. M1-to-M2 transition was also found in Li et al.'s study which applied an osteogenesis-inducing material, laponite (Lap), in bone defects. Although Lap is beneficial for bone regeneration, as a foreign object, it is still associated with inflammation. They found that MSCs converted laponite- (Lap-) induced M1 macrophages into the M2 phenotype, creating an anti-inflammatory/prosolving environment that promotes osteogenesis [26]. Nevertheless, the transplanted MSCs cannot be detected at 4 weeks posttransplantation, suggesting MSCs might regulate macrophage polarization during the early stage [23, 24].

MSC-induced M2 polarization is described in vitro as well. MSCs and macrophages cocultured with 1,25-dihydroxyvitamin D3 supplementation could reduce the secretion of inflammatory factors as a result of MSC-secreted PGE2 and VEGF. The CM from the cocultures further enhanced matrix maturation and mineralization of BMSCs under osteogenic conditions [27]. Preconditioning BMSCs with the combination of LPS and TNF-*α* was another strategy to affect macrophage polarization. Lin et al. found that PGE2 secreted from preconditioned BMSCs modulates M1 macrophages into an anti-inflammatory phenotype via the NF-*κ*B/COX2 pathway with no influence on mineralization [28]. In He et al.'s study, CM from MSCs cultured on LL-37-loaded silk fibroin nanoparticles (SFNPs) promotes M2 macrophage polarization. The increased IL-4 and TGF-*β*1 from MSCs cultured on LL-37-loaded SFNPs were regarded as the main cause of M2 polarization [29]. Anti-inflammatory cytokine IL-4 is beneficial for bone formation by enhancing scaffold vascularization and inhibiting osteoclast activation [30–33]. Excess IL-4 produced by genetically modified MSCs is another strategy to improve bone healing. IL-4-secreting MSCs are NF-*κ*B-responsive and continuously produce large amounts of IL-4 to further enhance M1-to-M2 transition. However, the IL-4-secreting MSCs reduced the osteogenic capacity in vitro, suggesting excessive IL-4 leaking into systemic circulation may potentially impair bone formation [34].

Both naïve MSCs and osteogenically differentiating MSCs are capable of altering the phenotypes of macrophages. After treatment with pre-osteoblast-derived exosomes, LPS-induced macrophages showed decreasing proinflammatory gene expression and lower levels of M1 markers. The authors realized that the differentiating MSC secretome could recruit more naïve MSCs to the injury site and produce a positive feedback loop to magnify naïve MSC exosome signals, thereby reducing subsequent inflammation and promoting bone regeneration [35].

In summary, MSC transplantation not only mitigates chronic inflammation but also promotes bone regeneration via M2 phenotype switching. Cotransplantation of MSCs could effectively ameliorate biomaterial-induced foreign body reactions in the bone that is associated with bone regeneration. Most noteworthy is the immunomodulatory effect of MSCs on macrophages. This provides a new insight that bone regeneration can be improved by osteoimmune environment modulation instead of enhancing bone formation through the direct regulation of osteolineage cells.

### 3.3. The Effects of Macrophages on MSC Osteogenesis

The skeletal and immune systems closely interact with each other by way of common cell precursors and molecular mediators. The different subtypes of macrophages and their influence on MSCs undergoing osteogenic differentiation are discussed in this section. In-depth details and results are listed in Tables 2 and 3.

#### 3.3.1. Bone Regeneration Enhanced by M1 Macrophages

Enhanced osteogenic differentiation of MSCs and bone regeneration have been observed in the proinflammatory environment, which is built by M1 macrophages. The macrophage cell line RAW 264.7 cultured with mesoporous silica nanospheres (MSNs) or graphene oxide (GO) increased the amount of proinflammatory cytokines (TNF-*α*, IL-6, IL-1*β*, and IFN-*γ*) and OSM. This inflammatory environment stimulated osteogenic differentiation of MSCs through OSM and NF-*κ*B pathways [36, 37]. Furthermore, Cu-MSN/macrophage CM upregulated OPG and downregulated RANKL in BMSCs to suppress osteoclastogenesis [36]. In coculture experiments, carbon nanohorn- (CNH-) engulfed macrophages also expressed OSM to accelerate osteogenic differentiation of MSCs via the STAT3 signaling pathway [38]. Lu et al. demonstrated that LPS-induced M1 macrophages promote osteogenesis via the COX2-PGE2 pathway. Increasing the ratio of M1 macrophages/MSCs in coculture to mimic the inflammatory reaction at the fracture site could further promote osteogenesis. However, OPG produced by MSCs was negatively regulated by LPS-induced M1 macrophages after coculture, suggesting the significance of the OPG-RANKL ratio and its relation to the role of M1 macrophages in modulating osteoclastogenesis need further investigation [39]. Tu et al. provided another perspective to explain the stimulatory effects of proinflammatory macrophages on MSC osteogenesis. IL-23 secretion from macrophages directly induced osteogenesis of MSCs by activating STAT3 and beta-catenin. Both calcium formation and ALP activity of MSCs were decreased when IL-23 in macrophage CM was neutralized by the IL-23 p19 antibody [40].

The effects of M1-to-M2 transition and the persistent proinflammatory status in bone healing have attracted extensive attention. Previous studies have shown that the injury-induced immune response at the proinflammatory stage is necessary for repair progress [32]. 1,25(OH)2D treatment during the inflammatory stage impeded fracture repair and suppressed M1 macrophages while promoting M2 macrophages. The M1-to-M2 transition caused by 1,25(OH)2D was accompanied by decreased release of osteogenic proteins such as OSM, TNF-*α*, and IL-6 from M1 macrophages. Overall, M1 macrophages are necessary and indispensable for the initiation of the proinflammatory phase during fracture repair [41]. The process of M1-to-M2 transition in a femur defect with LL-37-loaded SFNP Ti implants was demonstrated in He et al.'s study as well. The proinflammatory response of macrophages was largely induced in the injured site on day 4, but M1 macrophages began to decrease on day 7 gradually. The lower M1/M2 ratio after day 7 implies that the M1-to-M2 transition is necessary to improve osteointegration. Peptide LL-37 is more inclined to activate the M1 macrophages but is also capable of inducing anti-inflammatory responses in synergy with the microenvironment and other cytokines [29].

The precise timing of the M1-to-M2 transition for bone formation has been emphasized in the following study. Nathan et al. first utilized LPS-induced M1 macrophages to coculture with MSCs. IL-4 was then added for different durations to induce M2 phenotypes. The results suggest that a 72- to 96-hour proinflammatory environment is critical for appropriate MSC osteogenesis. Interestingly, the optimal time of the M1-to-M2 transition for MSC osteogenesis is gender-dependent. Such sex­linked difference in MSC osteogenesis might be explained by the different levels of steroid receptor expression, which mediates stem cell proliferation and differentiation [42].

#### 3.3.2. Bone Regeneration Enhanced by M2 Macrophages

Individual subtypes of macrophages lead to unique effects on MSCs. Here, we place greater emphasis on the proosteogenic effect of the M2 subtype, especially without any biomaterial involvement. In Gong et al.'s study, M2 macrophages enhanced osteogenic differentiation of MSCs, whereas M1 macrophages impaired it. Proregenerative cytokines, such as TGF-*β*, VEGF, and IGF-1, were produced by M2 macrophages, and detrimental inflammatory cytokines, such as IL-6, IL-12, and TNF-*α*, were produced by M1 macrophages and are the suspected mechanisms for the regulation of osteogenic differentiation [43]. However, in Zhang et al.'s study, M0 and M1 macrophages exclusively stimulate the osteogenic differentiation of MSCs in the early and middle stages via OSM and BMP2. In contrast, M2 macrophages are more beneficial to the mineralization of MSCs, the late stage of osteogenesis, in both the direct and indirect coculture systems [44]. He's team also clearly demonstrated how the macrophage subtypes engage in MSC osteogenesis. (1) M0 macrophages had a remarkable effect on promoting osteogenic differentiation. (2) M1 macrophages supported the proliferation of MSCs, while (3) M2 macrophages facilitated MSC osteogenesis. MSCs incubated with CM from M2 macrophages exhibited an enhanced capacity to form robust stem cell sheets [45]. Macrophages converted toward the M2 type by cytokine-preconditioned MSCs and IL-4-secreting MSCs were mentioned in Section 3.2 [28, 34]. Although both preconditioned MSCs and IL-4-secreting MSCs enhanced osteogenesis, there was a significant effect of timing in bone regeneration in vitro. After coculturing with macrophages, preconditioned MSCs promoted bone regeneration at an early stage (day 3), while IL-4-secreting MSC benefits occurred at a later stage (day 7). IL-4-secreting MSCs also possessed greater immunomodulatory capacity on M1-to-M2 transition based on the secretion of IL-4 and PGE2 [46].

#### 3.3.3. Bone Regeneration Enhanced by M2 Macrophages Collaborating with Biomaterials

Bone grafting with an implanted device is a general and promising surgical procedure when bone loss or a fracture has occurred. Besides providing structural stability to the injured site, bone substitutes further benefit osseointegration to its biocompatibility. However, increasing reports indicate that foreign implantation creates an inflammatory environment and forms fibrous capsules leading to negative effects on regeneration. To avoid the dilemma caused by the host-to-scaffold immune response, researchers optimize and improve the scaffolds using various strategies ameliorating the inflammatory environment to enhance the healing.

This section starts with macrophage subtypes triggered by physical factors directly and then addresses the indirect impact of the immune environment. Modifications of the surface properties are commonly being targeted to improve the performances of biomaterials [47, 48]. In Chen et al.'s study, the pore size of the nanoporous anodic alumina was the determinant of macrophage polarization. Compared with the polished material, the nanoporous structures inhibited the expression of proinflammatory cytokines and ROS and induced the shift toward an M2 phenotype. The porous alumina structure stimulated M2 macrophages to express a higher level of osteogenic-inducing factors (BMP2, BMP6, and WNT10b) and fibrosis-enhancing factors (TGF-*β*1 and VEGF), which are involved in the MSC osteogenesis [49]. Titanium (Ti) metal is widely used in clinical practice due to its remarkable osseointegration capacity. In the following two studies, the different nanostructured surface topographies on Ti that promote macrophage polarization are described. Wang et al. used different Ti specimens, including polished ones (P), ones with nanotubes (NTs) in small diameters (NT-30), and ones with NTs in large diameters (NT-100) to create a microenvironment for macrophage polarization. NT-100 induced M1 polarization and created a prohealing environment, while NT-30 induced M2 polarization, creating an anti-inflammatory environment. CM from NT-30-induced M2 macrophages enhanced MSC osteogenic differentiation [50]. Ma et al. fabricated superhydrophilic NT TiO_2_ surfaces with tube sizes of 30 and 80 nm via anodization at 5 and 20 V (denoted as NT5 and NT20, respectively). Macrophages cultured on NT5 and NT20 surfaces possessed different inflammatory behaviors. The M1 phenotype presented on NT20, whereas the M2 phenotype presented on NT5. NT surface topography and the respective CM acted together to promote the osteogenic behavior of MSCs in vitro. However, NT20-CM increased collagen synthesis and ECM mineralization of MSCs more than NT5-CM. In vivo, NT5 and NT20 both enhanced bone formation after 12 weeks postimplantation [51].

To mitigate the inflammation caused by the implanted materials, anti-inflammatory substances or drugs were applied together with the implanted scaffolds that locally modulated the immune environment. Iloprost, a prostacyclin (PGI2) analog with potent anti-inflammatory properties, was used in bone defects accompanied by a biphasic fibrin scaffold. Wendler's team found that iloprost leads to an increase of anti-inflammatory cAMP that suppresses M1 macrophages. The partial downregulation of inflammation improved bone regeneration outcomes of the mice [52]. The benefits of anti-inflammatory and proregenerative mediators and subsequent increases in M2 macrophages are mentioned in Zhu et al.'s and Yang et al.'s studies. Macrophages were first pretreated with Ti and crocin, an antioxidant and anti-inflammatory compound found in saffron, and then cultured with MSCs in the transwell system. Osteogenic differentiation of MSCs was enhanced due to the M2 polarization promoted by crocin. In addition, crocin polarized the M2 macrophages via the inhibition of p38 and c-Jun N-terminal kinase [53]. Lithium chloride (LiCl) was the selected drug to balance the Ti-induced inflammatory response in Yang et al.'s study. LiCl-derived M2 macrophage polarization and increases in anti-inflammatory and bone-related cytokines further promote MSC osteogenesis [54].

Biomaterials possess unique characteristics that contribute to different immunomodulatory properties and are capable of shaping the local environment as well. Hierarchical intrafibrillar mineralized collagen (HIMC) and strontium-incorporated calcium silicate (Sr-CS) were used in scaffolds to enhance bone regeneration by promoting M2 polarization in vitro and in vivo [55, 56]. HIMC facilitated M2 macrophage polarization and IL-4 secretion to promote MSC osteogenesis. In critical-sized mandible defect models, host MSCs were recruited to the HIMC-loaded IL-4 implantation site and promoted bone regeneration within the anti-inflammatory environment built by HIMC [55]. Similar results were found in Wang et al.'s study; extracts from Sr-CS-pretreated macrophages not only suppressed the inflammatory response but also facilitated MSC osteogenesis and chondrogenesis in vitro. Osteochondral regeneration was significantly improved by Sr-CS in vivo [56]. Calcium phosphates (CaPs), a kind of bone graft material, were applied in the LPS-stimulated macrophage system. CaPs reversed the inflammatory condition caused by LPS-stimulated macrophages, evidenced by the dramatically increased anti-inflammatory-related genes. Osteoclastic-related genes also decreased. The microenvironment created after culturing macrophages on CaPs showed more potent osteogenic effects, fostering osteogenic differentiation of both BMSCs and SaOS-2 cells [57]. ECM bioscaffolds elicited contradictory macrophage phenotypes in Wu et al.'s study. ECM particles had a greater tendency to induce macrophages toward M1 polarization, while ECM gels were more inclined to promote M2 polarization. Although surgical transplantation of ECM particles and ECM gels both showed a better healing tendency in periodontal wounds compared with the control group, the ECM gels showed notable improvements which were attributed to M2 polarization. Notch, PI3K/Akt, integrin, and MEK/ERK are possible signaling pathways responding to the various ECM hydrogels to influence macrophage polarization [58]. Gao et al. performed whole-genome expression analysis to create a map of macrophages that are regulated by biomaterials. Functionalized polyetheretherketone (PEEK) surfaces not only inhibited early proinflammatory M1 polarization but also facilitated M2 differentiation. MSC osteogenesis was promoted after being cultured with the macrophage CM collected from the PEEK surfaces. Inhibited osteoclastogenesis was evidenced by decreased TRAP activity in the macrophages cultured on PEEK surfaces. Thus, enhanced osteogenesis and suppressed osteoclastogenesis synergistically facilitated peri-implant osseointegration. The whole-genome expression analysis of the macrophages was performed after culturing on PEEK for 3 days. The toll-like receptor (TLR), NOD-like receptor (NLR) signaling pathway, and focal adhesion were downregulated, eventually assembling into downstream MAPK and NF-*κ*B signaling cascades to bring about reduced transcription of inflammation-related genes (NOS2, COX2, MIP-1*α*/*β*, and CSF1/2). TNF-*α* and JAK-STAT signaling pathways were also inhibited. Consequently, the autocrine response of macrophages led to an attenuating feedback loop that mitigated the acute inflammatory reaction [59].

#### 3.3.4. Bone Regeneration Inhibited by Macrophages

Although most of the literature shows that macrophages positively benefit MSC osteogenesis, some studies conclude that macrophages inhibit osteogenesis. In Tang et al.'s study, polarized macrophages (M1 or M2) and MSCs formed 3D spheroids at a ratio of 1 to 1 via centrifugation. These 3D spheroids were placed in an osteogenic induction medium for 28 days, and then they examined the degree of osteogenic differentiation. Both subtypes of macrophages inhibited the osteogenic differentiation of MSCs, with M2 macrophages exhibiting an even stronger inhibiting effect than M1 macrophages. N-cadherin was considered the mediator between macrophages and MSCs responsible for the inhibition of osteogenesis [60]. Another study published from the same team followed the same (3D) coculture methods but with poly(lactic-co-glycolic) acid/polycaprolactone scaffolds demonstrating similar results. Downregulated secretion of OSM and bone morphogenetic protein 2 (BMP2) was observed in the macrophage-MSC cocultures. The gene expression levels of osteogenic markers (ALP, BSP, and RUNX2) were inhibited as well [61]. Multiple factors such as the source of stem cells, polarization strategies for macrophages, and cell ratios are possible explanations for this inhibited osteogenesis. However, the majority of the selected studies in this review support the enhancement of osteogenic differentiation by macrophages. The mechanism behind this phenomenon needs further confirmation and more evidence from rigorous studies.

In summary, macrophages indeed regulate the bone microenvironment to enhance bone healing though the effects of various macrophage subtypes are still under debate. A major proportion of the selected studies demonstrated that M2 macrophages account for the improvement of bone regeneration by both enhancing MSC osteogenesis and repressing inflammation. Biomaterial surface topography could trigger different morphological alterations of macrophages by affecting focal adhesion formation and cytoskeletal structure. The profiles of cytokines released from different subtypes of macrophages promote regeneration at different stages of bone repair. On the other hand, retroregulative cytokines released by stimulated MSCs provide a groundwork for systematically elucidating the likely mechanism and potential targets for enhancing osseointegration. In conclusion, the process and timing of M1-to-M2 transition and its subsequent effects are essential for bone regeneration.

## 4. Discussion

The field of osteoimmunology started by investigating the effect of the immune system on bone, yet the two decades of osteoimmunology witnessed the emerging role of the skeletal system in the regulation of the immune system, emphasizing the inseparable link between them [62]. The concept of mutual dependency of the two systems must be considered when exploring disease mechanisms or designing therapeutic strategies wherever the skeletal and/or immune systems are involved. Thanks to our improved understanding of osteoimmunology, clinicians can use drugs classically used for osteoporosis to treat immunological (e.g., denosumab for RA). As our understanding progresses and the crosstalk between the two systems is elucidated, they may start looking like a single system [63].

Interaction between MSCs and macrophages has been well established. MSCs have been widely investigated for treating various pathologies with marked inflammation—such as spinal cord injuries—and have shown great anti-inflammatory properties resulting in better outcomes [64]. In vitro and in vivo preclinical studies have shown the essential crosstalk between MSCs and tissue macrophages [65]. Increased understanding of this crosstalk would improve understanding of the immunomodulatory capacity of MSCs and inform the development and testing of potential mechanisms of action to improve therapeutic use of MSCs in treating diseases [66].

While there has already been a review written on the same topic [11], a systematic review has several advantages. By compiling all relevant studies on a particular topic, there is less likely to be biased and we can establish whether findings are consistent and generalizable, which helps clarify current understanding and future directions for readers. Readers can also gauge our review process individually as our protocol is transparent at each phase of the synthesis process [67]. There is a systematic review already published on the effect of MSC secretions on macrophages which is distinct from our systematic review [68]. While we also look at the effect of MSC secretions on macrophages, we further consider the effects of MSCs and macrophages on bone regeneration. As shown in Figure 2, many more papers have been published in the past 3 years about this topic, which shows an increasing relevance and importance in understanding the role of MSCs and macrophages in healing.

MSCs are known to promote polarization of monocytes and macrophages toward the anti-inflammatory (type 2) phenotype and directly inhibit differentiation into the type 1 phenotype and dendritic cells by secreting interleukin-1 receptor antagonist (IL-1RA). Anti-inflammatory monocytes secrete high levels of IL-10, which is crucial for the beneficial effects of MSCs and results in a positive feedback loop of inducing monocyte differentiation toward the anti-inflammatory phenotype [12]. From our systematic review, we found that MSCs induce M2 macrophages, consistent with findings in previous studies. With the increasing relevance of cell therapy, the anti-inflammatory and immunomodulatory nature of MSCs through M2 macrophages makes MSCs an attractive therapeutic option for many diseases [69]. MSC-mediated macrophage polarization has been shown to be beneficial in a myriad of conditions ranging from traumatic spinal cord injury to tendon rupture to dilated cardiomyopathy [70].

Most of our selected studies suggest that M2 macrophages are more important in osteogenesis while M1 macrophages play a minor role. However, some of the selected studies found that M1 macrophages enhanced bone regeneration. These contradictory results can be explained by different subtypes of macrophages exerting unique functions during their respective stages of the healing process. The contribution of M1 and M2 macrophages in fracture healing is sequential and equally important [71]. Classically activated M1 macrophages are inflammatory and further secrete IL-1, IL-6, TNF-*α*, MCP-1, and MIP-1 to maintain the recruitment of monocytes. They perform phagocytosis to remove necrotic cells as well as the fibrin thrombus formed during healing. Alternatively, activated M2 macrophages are anti-inflammatory and are found more commonly in the later stages of inflammation as they promote tissue repair through IL-10, TGF-beta, BMP2, and VEGF. Their role is to recruit mesenchymal progenitor cells, induce osteochondral differentiation, and prompt angiogenesis.

Despite the proinflammatory effect of M1 macrophages, they are still necessary for the process of healing [5, 8]. In mouse models of acute pancreatitis, depleting macrophages immediately after the acute inflammatory response significantly reduced duct-like structures. This indicates that M1 macrophages play a key role in acinar-ductal metaplasia which is necessary for healing [72]. Other models also found M1 macrophages critical as depleting macrophages eliminated the benefits of therapeutics that promote M2 differentiation [73]. Although M1 macrophages are necessary for the healing process, their presence over a long period of time was detrimental. Osteoarthritis is associated with an elevated ratio of M1-to-M2 macrophages in peripheral blood. The patients with the higher ratio of M1-to-M2 macrophages in synovial fluid correlated with the more severe osteoarthritis symptom [74].

Classification of M1 or M2 macrophages is normally based on specific markers that tend to be associated with either M1 or M2. M2 macrophages have subclassifications, some of which include markers that have been traditionally considered M1 markers. M2 terminology covers a functionally diverse group of macrophages rather than a uniform activation [75]. Unlike T cells, which undergo extensive epigenetic modifications during differentiation, macrophages retain their plasticity and are responsive to environmental signals. Relying on a single marker to identify a macrophage population can be problematic [76]. Based on this understanding of macrophage classification, we can understand why different studies have different findings regarding the role of M1 and M2 macrophages in promoting MSC osteogenic differentiation. The authors only used a few cell surface markers to classify macrophages, and while it simplifies the process of classification, we find it insufficient in understanding the role of macrophages in bone healing as different macrophages show varying degrees of participation throughout the process.

Among our selected studies, the NF-*κ*B and OSM signaling pathways are most commonly referenced as the mechanisms most likely responsible for the observed interactions between macrophages and MSCs. NF-*κ*B has long been considered a prototypical proinflammatory signaling pathway that regulates multiple aspects of innate and adaptive immune functions and serves as a pivotal mediator of inflammatory responses [77]. The proinflammatory cytokines driven by NF-*κ*B are powerful modulators of osteoblast and osteoclast activity. Activation of NF-*κ*B is also crucial for osteoclast differentiation and activation. These characteristics suggest the great potential of NF-*κ*B as a therapeutic target for treating inflammation-associated bone disorders. The effects of NF-*κ*B in osteoblasts are not as clear but have been reported to repress osteoblast differentiation as well as a prosurvival role in osteoblastic cells [78, 79]. Oncostatin M (OSM) belongs to the IL-6 family of cytokines and is associated with multiple biological processes and cellular responses, including growth, differentiation, and inflammation [80]. OSM displays anabolic effects on cortical and trabecular while also driving osteoclast formation. Recruitment of STAT3 or MAPK1/2 by OSM initiated remodeling in conditions like arthritis and osteoporosis and aided in the repair of fractures [81]. OSM stimulates osteoclasts by inducing osteoblastic expression of RANKL, which is mediated by the OSM receptor (OSMR):gp130 receptor complex and downstream initiation of JAK/STAT signaling (namely, STAT3) within osteoblasts [82]. Based on our understanding of this mechanism, macrophage-secreted OSM regulates MSCs and bone cells, which directly impacts the bone remodeling process.

The high regenerative capacity in bone means that most injuries heal well without intervention. Despite this, large defects caused by tumor resections and severe nonunion fractures cannot regenerate properly and require surgery. Currently, the gold standard is autografting but it is limited mainly by its short supply and the morbidity associated with harvesting [83]. Biomaterials are an attractive alternative that can provide the structure necessary for regeneration without the limitations of autografting. These biomaterials were initially “bioinert,” but now, many of them are intentionally “bioactive” to augment the healing process. These materials typically consist of bioactive ceramics, bioactive glasses, biologic or synthetic polymers, or composites of the above [84]. However, inflammatory responses occur when these foreign biomaterials are implanted, leading to a cascade of cellular reactions [85]. Neutrophils are responsible for producing inflammatory mediators that promote macrophages differentiating into M1 and M2. If acute inflammation is not resolved, biomaterial-adherent M1 macrophages will begin to form giant cells and transition into chronic inflammation [86]. There is a wide range of treatments to reduce inflammation, but many systemic treatments cannot achieve an adequate local concentration and may have significant adverse effects. Therefore, incorporating anti-inflammatory molecules into solid scaffolds of biomaterials is attractive. Many different molecules capable of reducing inflammation are at various stages of testing. These molecules most commonly target inflammatory cytokines to optimize macrophage polarization [87]. Among the selected studies related to biomaterials, there is substantial evidence that inflammation can be reduced by modulating macrophage polarization. While there are many studies investigating treatments that directly promote healing or affect MSCs to augment healing, we excluded these studies as our systematic review focuses on the relationship between macrophages and MSCs in bone regeneration, and these are not strictly relevant [88–90].

## 5. Conclusion

The demand for realizing the interaction between MSCs and other cells has soared since transplantation of MSCs is considered a beneficial therapeutic strategy in regenerative medicine. As bone metabolism is tightly regulated by the immune system, macrophages have been drawing attention for their immunomodulatory and osteogenic potential in fracture healing. The crosstalk between MSCs and macrophages during bone regeneration is systematically described in this review. The key points about the crosstalk between these two cells can be roughly divided into two major categories: (1) the effects of transplanted MSCs on macrophage phenotype switching and (2) how the subtypes of macrophages influence endogenous MSC osteogenesis. MSC transplantation improves bone regeneration and is accompanied by macrophage M2 phenotype switching. Transplanted MSCs and M2 macrophages together create a proresolving environment by enriching specific anti-inflammatory cytokines and osteogenic-inducing factors. Furthermore, M2 macrophages possess great potential for accelerating bone healing in comparison with M0 and M1 macrophages. This review provides compelling evidence that the crosstalk between MSCs and macrophages enhances their regenerative potential on bone via unique secretomes. The phenotype switching time frame of macrophages orchestrates that the microenvironment is crucial for bone regeneration. This review also highlights spatiotemporal changes in the immune system during bone hemostasis. Comprehensive investigations between MSCs and macrophages can extend to other bone diseases and can be beneficial in the clinical application of MSC- or macrophage-based therapies.

## Figures and Tables

**Figure 3:**
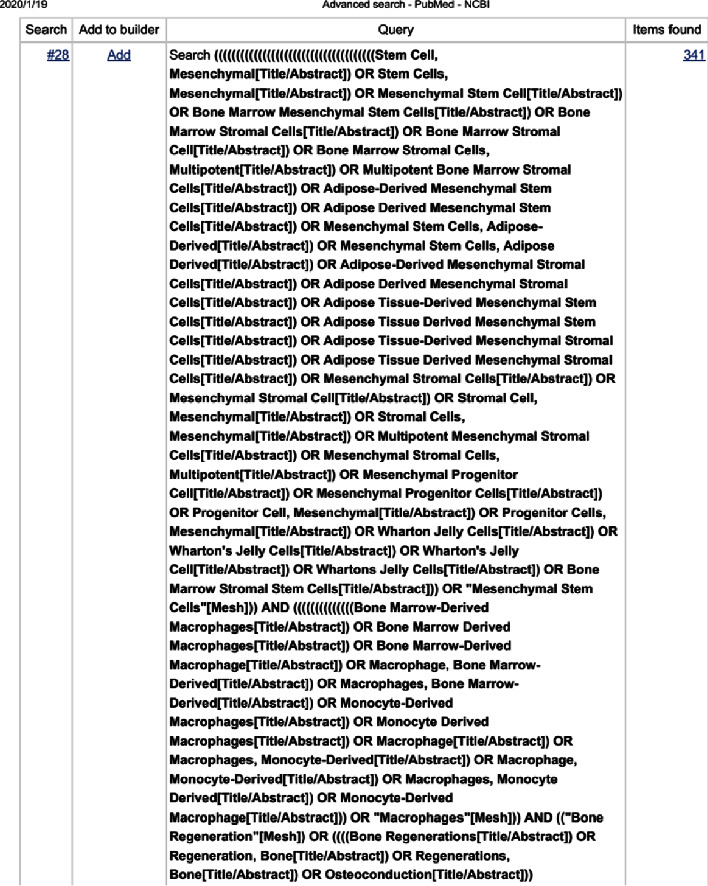


**Figure 4:**
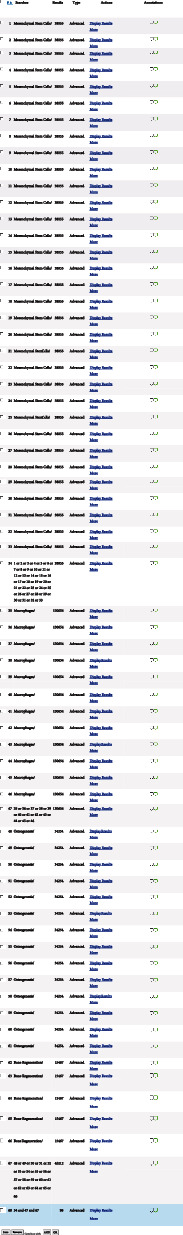


**Table 1:** Immunoregulatory potential of MSCs on macrophages in bone regeneration.

Author	Cell source	Study type	Cell management	Immunoregulatory potential of MSCs on M*φ*s	Proposed mechanisms
Tasso R 2013	C57BL/6 mice—BMSCs; C57BL/6 mice—M*φ*s	In vitro & in vivo	In vitro: M*φ*s cultured in the IL-1a-stimulated BMSC-CM In vivo: BMSCs seeded on bioceramic scaffolds are transplanted	In vitro: the percentage of M2 M*φ*s significantly increases after M*φ*s cultured in the CM from BMSCs In vivo: implanted BMSCs induce M*φ* switching to a proresolving phenotype and recruit vasculogenic and osteogenic progenitors from BM	PGE2 secreted from BMSCs activates the NF-*κ*B pathway to affect M2 M*φ* polarization
Seebach E 2014	SD rats—BMSCs; SD rats—M*φ*s	In vivo	BMSCs embedded in a fibrin carrier are implanted into femoral bone defects	BMSC composites attract proinflammatory M1 M*φ*s and endothelial progenitors and then promote implant integration, angiogenesis, and tissue maturation	/
Tour G 2014	Lewis GFP transgenic rat—BMSCs; SD rats—M*φ*s	In vivo	BMSCs with HA-ECM are implanted into calvarial bone defects	M1 M*φ*s were prevalent than M2 M*φ*s in the calvarial defects at 2 weeks after surgery	/
Lin T 2017	C57BL/6 mice—BMSCs; C57BL/6 mice—M*φ*s	In vitro	M*φ*s are treated with the CM from LPS-exposed MSC^NF-*κ*BREIL4^	CM from MSC^NF-*κ*BREIL4^ modulates inflammatory M1 M*φ*s into an anti-inflammatory M2 M*φ*s	NF-*κ*B-sensing MSC^NF-*κ*BREIL4^ produces excessive IL-4 for immunomodulation
Lin T 2017	C57BL/6 mice—BMSCs; C57BL/6 mice—M*φ*s	In vitro	Preconditioned BMSCs with LPS plus TNF-*α* culture with M1 M*φ*s	Preconditioned BMSCs modulate M1 M*φ*s into an anti-inflammatory phenotype and increase PGE2 production but not affect mineralization	Preconditioned BMSC-secreted PGE2 can be stimulated by TNF-*α* through the NF-*κ*B/COX2-dependent pathway
Saldana L 2017	Human—BMSCs; THP-1—M*φ*s	In vitro	BMSCs undergo osteogenic differentiation with the CM from the cocultures of BMSCs, M*φ*s, and 1,25D3	1,25D3 promotes the switching of cocultured M*φ*s toward the M2 phenotype secreting anti-inflammatory factors (IL-10, PGE2) to enhance matrix maturation and mineralization of BMSCs	/
Li T 2018	SD rats—BMSCs; RAW 264.7—M*φ*s	In vitro & in vivo	In vitro: Lap/M*φ* CM with osteogenic components is applied to stimulate BMSCs In vivo: Lap+BMSCs are injected into the bone defect	In vitro: BMSCs reversed M1 M*φ*s induced by Lap into M2 M*φ*s and promoted osteogenesis In vivo: the Lap+BMSC group shows obvious new bone formation with a significant increase in M2 M*φ*s	Activation of the OSM pathway is likely involved in the enhanced osteogenesis by BMSCs
He Y 2019	SD rats—BMSCs; RAW 264.7—M*φ*s	In vitro *&* in vivo	In vitro: CM from BMSCs seeded on Ti-SF/LL-37 is applied on M*φ* culturing In vivo: LL-37-loaded SFNPs of Ti rods are inserted into the bone defect	In vitro: M2 phenotype switching of M*φ*s is induced by the BMSCs seeded on Ti-SF/LL-37 In vivo: demonstrated in Table 2	/
Wei F 2019	Human—BMSCs; RAW 264.7—M*φ*s	In vitro	LPS-induced M*φ*s are treated with exosomes first isolating from osteogenically differentiating BMSCs	The uptake of exosomes significantly decreases the M1 phenotypic marker of LPS-induced M*φ*s	/

BMSCs: bone marrow stem cells; M*φ*s: macrophages; CM: conditioned medium; PGE2: prostaglandin E2; NF-*κ*B: nuclear factor-kappa B; GFP: green fluorescent protein; HA-ECM: hydroxyapatite-extracellular matrix; LPS: lipopolysaccharide; TNF-*α*: tumor necrosis factor-alpha; COX2: cyclooxygenase 2; 1,25D3: 1,25-dihydroxyvitamin D3; Lap: laponite; OSM: oncostatin M; Ti-SF: titanium-silk fibroin; SFNPs: silk fibroin nanoparticles.

**Table 2:** Involvement of M1 macrophages in MSC osteogenic differentiation and bone regeneration.

Author	Cell source	Study type	Cell management	Involvement of M*φ* polarization in MSC osteogenic induction	Proposed mechanisms
Tu B 2015	Human—BMSCs; THP-1—M*φ*s	In vitro	M*φ* CM collected for treating BMSCs under osteogenic induction conditions	IL-23 secretion from proinflammatory M*φ*s promotes the osteogenesis of BMSCs	M*φ*-secreted IL-23 activates the STAT3 and *β*-catenin signaling and thus promotes the osteogenic differentiation of BMSCs
Hirata E 2016	Human—BMSCs; human—M*φ*s	In vitro	Coculture of BMSCs and M*φ*s in the presence of CNHs	ALP activity is increased under the coculture of M*φ*s and MSCs in the presence of CNHs	OSM from activated M*φ*s induces osteoblast differentiation and matrix mineralization through STAT3
Shi M 2016	Human—BMSCs; RAW 264.7—M*φ*s	In vitro	BMSCs cultured in Cu-MSN/M*φ* CM under osteogenic differentiation	M*φ*s phagocytize Cu-MSNs and produce proinflammatory cytokines leading to better osteogenic differentiation of BMSCs	Cu-MSN/M*φ* CM enhances the osteogenic differentiation of BMSCs through the activation of the OSM pathway
Lu LY 2017	C57BL/6 mice—BMSCs; C57BL/6 mice—M*φ*s	In vitro	Coculture of BMSCs and polarized M*φ*s (M1 induced by LPS and M2 induced by IL-4)	Polarized M*φ*s enhance bone mineralization, especially proinflammatory M1 M*φ*s	M1 M*φ*s enhance BMSC osteogenesis and bone formation via the COX2-PGE2 pathway
Tang H 2017	Human—ADSCs; THP-1—M*φ*s	In vitro	3D spheroid cocultures of M1 M*φ*s and ADSCs are conducted under osteogenic differentiation conditions	The osteogenic differentiation of ADSCs is inhibited by M1 M*φ*s	N-cadherin-mediated cell-cell interactions between M1 M*φ*s and ADMSCs result in inhibited osteogenesis
Xue D 2018	Human—BMSCs; RAW 264.7—M*φ*s	In vitro	BMSCs undergo osteogenic differentiation added with the CM from GO/M*φ*s	Coculture of GO and M*φ*s induced M1 M*φ* transition and produced proinflammatory cytokines in the CM, further enhancing BMSC osteogenesis	The proinflammatory environment induced by GO promote osteogenic differentiation of BMSCs through OSM and NF-*κ*B pathways
Wasnik S 2018	C57BL/6 mice—BMSCs; C57BL/6 mice—M*φ*s	In vivo	Mice with the fracture at the midshaft receive a daily s.c. dose of 1,25(OH)2D	The suppression of fracture healing induced by 1,25(OH)2D is mediated by the inhibition of M1 M*φ*s during the proinflammatory stage	/
Nathan K 2019	C57BL/6 mice—BMSCs; C57BL/6 mice—M*φ*s	In vitro	Coculture of BMSCs and M1 M*φ*s in the presence of IL-4 under the osteogenic induction medium	Temporal modulation of M1-to-M2 polarization maximizes MSC matrix mineralization	/
Tang H 2019	Human—ADSCs; THP-1—M*φ*s	In vitro	M1 M*φ*s and ADSC coculture on PLGA/PCL scaffolds with osteogenic induction components	M1 M*φ*s inhibit the osteogenic differentiation of ADMSCs on 3D PLGA/PCL scaffolds	M1 M*φ*s inhibit osteogenic-related pathways (BMP & OSM signaling) during ADSC differentiation
Y He 2019	SD rats—BMSCs; RAW 264.7—M*φ*s	In vitro *&* in vivo	In vitro: CM from M*φ*s seeded on Ti-SF/LL-37 is applied on BMSC culturing In vivo: LL-37-loaded SFNPs of Ti rods are inserted into the bone defect	In vitro: osteogenic differentiation of BMSCs was enhanced by additional CM from M*φ*s incubated on Ti-SF/LL-37 In vivo: The Ti-SF/LL-37 group effectively induced both proinflammatory factors and exhibited improved osteogenesis ability	/

BMSCs: bone marrow stem cells; M*φ*s: macrophages; CNHs: carbon nanohorns; ALP: alkaline phosphatase; OSM: oncostatin M; STAT3: signal transducer and activator of transcription 3; Cu-MSNs: Cu-containing mesoporous silica nanospheres; CM: conditioned medium; COX2: cyclooxygenase 2; PGE2: prostaglandin E2; GO: graphene oxide; NF-*κ*B: nuclear factor-kappa B.

**Table 3:** Involvement of M2 macrophages in MSC osteogenic differentiation and bone regeneration.

Author	Cell source	Study type	Cell management	Involvement of M*φ* polarization in MSC osteogenic induction	Proposed mechanisms
Gong L 2016	C57BL/6 mice—BMSCs; C57BL/6 mice—M*φ*s	In vitro	Coculture of BMSCs and polarized M*φ*s (M1 induced by LPS and M2 induced by IL-4) with the osteogenic medium	M2 M*φ*s enhance osteoblast differentiation of MSCs	Proregenerative cytokines (TGF-*β*, VEGF, and IGF-1) produced by M2 M*φ*s facilitate MSC osteogenesis
Chen Z 2017	SD rats—BMSCs; RAW 264.7—M*φ*s	In vitro	CM from nanopore structure/M*φ*s is applied to stimulate BMSCs under the osteogenic induction medium	Osteogenesis of BMSCs is enhanced by the stimulation of the nanostructure/M*φ* CM	Osteogenic pathways (Wnt and BMP) of BMSCs are regulated by different nanopore-induced inflammatory environments
Zhang Y 2017	Human—ADSCs; THP-1—M*φ*s	In vitro	Direct and indirect coculture of ADSCs and polarized M*φ*s during osteogenic differentiation (M1 induced by IFN-*γ* & LPS and M2 induced by IL-4 & IL-13)	M2 M*φ*s have beneficial effects on ADSC mineralization by promoting their proliferation and osteogenic differentiation	M2 M*φ*s enhance osteogenic differentiation of MSCs in a manner dependent on OSM and BMP2 signaling pathways
Tang H 2017	Human—ADSCs; THP-1—M*φ*s	In vitro	3D spheroid cocultures of M2 M*φ*s and ADSCs are conducted under osteogenic differentiation conditions	The osteogenic differentiation of ADSCs was inhibited by M2 M*φ*s	N-cadherin-mediated cell-cell interactions between M2 M*φ*s and ADMSCs result in inhibited osteogenesis
He XT 2018	C57BL/6 mice—BMSCs; RAW 264.7—M*φ*s	In vitro	BMSCs incubated with different CMs generated by unpolarized M*φ*s (M0) or polarized M*φ*s (M1 and M2) supplemented with osteoinductive media	CM from M2 M*φ*s exhibits the potential to foster osteogenic differentiation of BMSCs	/
Wang J 2018	C57BL/6 mice—BMSCs; C57BL/6 mice—M*φ*s	In vitro	BMSCs undergo osteogenic differentiation with NT/M*φ* CM	NT-30 induces more M2 M*φ*s while enhancing BMSC osteogenesis while NT-100 induces M1 M*φ* polarization	/
Ma QL 2018	Human—BMSCs; human—M*φ*s	In vitro & in vivo	In vitro: osteogenic differentiation of BMSCs on different Ti surfaces in CM from M*φ*s In vivo: three types of Ti implants inserted in the distal femur	In vitro: the NT surfaces and corresponding CM types together promote osteogenic gene expression in BMSCs, and osteoclast formation is likely promoted by factors (sRANKL, OPG, and M-CSF) secreted by BMSCs cultured in NT20-CM but suppressed in NT5-CM In vivo: the NT5 and NT20 surfaces lead to enhanced bone formation after 12 weeks postimplantation	NF-*κ*B and BMP pathways activated by the polarized macrophages are involved in both osteogenesis and osteoclastogenesis
Jin SS 2019	Human—BMSCs; THP-1—M*φ*s	In vitro & in vivo	In vitro: BMSCs are cultured with supernatants of M*φ*s seeded on scaffolds In vivo: deplete the M*φ*s by clodronate liposomes and implant HIMC as a bone graft in rat mandible defect models	In vitro: M2 M*φ* polarization induced by HIMC interacts with BMSCs to promote osteogenic differentiation and mineralization In vivo: the ectopic bone formation stimulated by tricalcium phosphate is blocked by M*φ* depletion	HIMC intrinsically promotes M2 M*φ* polarization with IL-4 secretion, further enhancing BMSC osteogenesis
Sadowska JM 2019	Human—BMSCs, human—SaOS-2; RAW 264.7—M*φ*s	In vitro	LPS-stimulated M*φ*s first cultured on the CaPs and CaP-M*φ*-conditioned extracts are incubated with the bone-forming cells (BMSCs and SaOS-2) for osteogenic stimulation	The microenvironment created after culturing M*φ*s on CDHA showed more potent osteogenic effects, fostering osteogenic differentiation of both BMSCs and SaOS-2 cells	/
Tang H 2019	Human—ADSCs; THP-1—M*φ*s	In vitro	M*φ*s (M1, M2) and ADSC coculture on PLGA/PCL scaffolds with osteogenic induction components	Both macrophage subtypes inhibit the osteogenic differentiation of ADMSCs on 3D PLGA/PCL scaffolds	M*φ*s inhibit osteogenic-related pathways (BMP & OSM signaling) during ADSC differentiation
Yang C 2019	Wistar rats—BMSCs; RAW 264.7—M*φ*s	In vitro *&* in vivo	In vitro: BMSCs undergo osteogenesis under the CM collected from M*φ*s stimulated by Ti+LiCl In vivo: the air pouch models are injected with Ti+LiCl	In vitro: LiCl promotes M2 polarization, and the better osteogenic differentiation driven by Ti+LiCl-stimulated CM was also observed In vivo: the LiCl group has fewer infiltrating cells, and thinner fibrous layers further induce higher levels of anti-inflammatory cytokines from M2	LiCl attenuated wear Ti particle-induced inflammation via the suppression of ERK and p38 phosphorylation
Zhu K 2019	C57BL/6 mice—BMSCs; RAW 264.7—M*φ*s	In vitro *&* in vivo	In vitro: crocin-pretreated M*φ*s indirectly cocultured with BMSCs In vivo: the air pouch model is treated with Ti particles+crocin	In vitro: crocin-pretreated M*φ*s provide an immunomodulatory microenvironment that further promotes osteogenic differentiation In vivo: crocin inhibits Ti particle-induced inflammation and induces M2 polarization	M2 polarization promoted by crocin via the inhibition of p38 and c-Jun N-terminal kinase
Lin T 2019	Balb/c mice—BMSCs; Balb/c mice—M*φ*s	In vitro	Coculture of BMSCs (preconditioned or genetically modified IL-4-secreting BMSCs) and M*φ*s directly under the osteogenic medium, including LPS-contaminated polyethylene particles	Both IL-4-secreting BMSCs and preconditioned BMSCs enhance osteogenesis during coculture but at different stages (preconditioned MSCs on day 3 and IL-4-secreting MSCs on day 7)	Enhanced osteogenesis at a later stage associated with the M1-to-M2 M*φ* transition
Wang C 2019	NZW rabbits—BMSCs; RAW 264.7—M*φ*s	In vitro	Osteogenic differentiation of BMSCs with the supernatants of CS- and Sr-CS-pretreated M*φ*s	Extracts from M*φ*s cultured in Sr-CS promote M*φ* polarization and enhance BMSC osteogenesis	/
Wendler S 2019	C57BL/6 mice—BMSCs; C57BL/6 mice—M*φ*s	In vitro & in vivo	In vitro: osteogenic differentiation of BMSCs treated with the CM from bone marrow cells and iloprost In vivo: implantation of a biphasic fibrin scaffold with iloprost into the bone defect	Iloprost decreases the proinflammatory phase and enhances the anti-inflammatory phase to improve bone healing In vivo: postsurgery of receiving iloprost shows an improved fracture healing outcome of the mice	Iloprost signaling leads to an increase of anti-inflammatory agent cAMP to suppress M1
Wu RX 2019	SD rats—BMSCs; SD rats—M*φ*s	In vivo	Rat periodontal defects are implanted with ECM particles and gels	Gel-type bone ECM has a greater tendency toward M2 polarization showing a better healing tendency	/
Gao A 2020	Human—BMSCs; THP-1—M*φ*s	In vitro & in vivo	In vitro: BMSCs undergo osteogenic differentiation with M*φ* CM collected from the PEEK culture system (rinsing in pH 1.8) In vivo: PEEK (rinsing in pH 1.8) is implanted in the bone defect on the rat femur	In vitro: M*φ*s in contact with PEEK expressing the M2 phenotype create a more favorable microenvironment for osteogenic differentiation of BMSCs In vivo: the quality and quantity of newly formed bone surrounding the pH 1.8 implants better than the PEEK and O_2_ groups	PI3K-Akt signaling, TLR signaling, NLR signaling, and TNF-*α* signaling all are the mechanisms that alleviate the acute inflammatory response and indirectly enhance osteogenesis

BMSCs: bone marrow stem cells; M*φ*s: macrophages; LPS: lipopolysaccharides; TGF-*β*: transforming growth factor-beta; VEGF: vascular endothelial growth factor; IGF-1: insulin-like growth factor 1; CM: conditioned medium; BMP: bone morphogenetic protein; ADSCs: adipose-derived stem cells; IFN-*γ*: interferon-gamma; OSM: oncostatin M; NT: nanotube; Ti: titanium; sRANKL: soluble receptor activator of nuclear factor-kappa B ligand; OPG: osteoprotegerin; M-CSF: macrophage colony-stimulating factor; NF-*κ*B: nuclear factor-kappa B; HIMC: hierarchical intrafibrillar mineralized collagen; CaPs: calcium phosphates; CDHA: calcium-deficient hydroxyapatite; PLGA/PCL: poly(lactic-co-glycolic) acid/polycaprolactone; Ti+LiCl: titanium+lithium chloride; Sr-CS: strontium-incorporated calcium silicate; cAMP: cyclic adenosine monophosphate; ECM: extracellular matrix; PEEK: polyetheretherketone; PI3K-Akt: phosphoinositide 3-kinase/protein kinase B; TLR: toll-like receptor; NLR: NOD-like receptor; TNF-*α*: tumor necrosis factor-alpha.

**Table 4:** 

Section and topic	No.	Quality criteria	Yes	No
Title/keywords/introduction	1	Were the study hypothesis/aim/objective being clearly described		
Method	2	Were the experimental design for the study being well described		
	3	Were the method and materials being well described		
	4	Were the time points of data collection being clearly defined		
	5	Were the main outcome measurements being clearly defined		
	6	Were the experimental group being well compared with the control group		
Discussion	7	Were the results being well described		
	8	Were the limitation of the article being discussed		

Wells and Littell [22].

**Table 5:** 

Study	1	2	3	4	5	6	7	8	Quality score
Tasso et al.	Yes	Yes	Yes	Yes	Yes	Yes	Yes	No	7
Seebach et al.	Yes	Yes	Yes	Yes	Yes	Yes	Yes	Yes	8
Tour et al.	Yes	Yes	Yes	Yes	No	Yes	Yes	No	6
Tu et al.	Yes	Yes	Yes	Yes	No	Yes	Yes	Yes	8
Gong et al.	Yes	Yes	Yes	Yes	No	No	Yes	No	5
Hirata et al.	Yes	Yes	Yes	Yes	No	Yes	Yes	No	6
Shi et al.	Yes	Yes	Yes	Yes	Yes	No	Yes	No	6
Chen et al.	Yes	Yes	Yes	Yes	Yes	Yes	Yes	No	7
Lin et al. (Cytotherapy)	Yes	Yes	Yes	Yes	Yes	Yes	Yes	No	7
Lin et al. (Stem Cell Res Ther)	Yes	Yes	Yes	Yes	Yes	Yes	Yes	No	7
Lu et al.	Yes	Yes	Yes	Yes	Yes	Yes	Yes	Yes	8
Saldana et al.	Yes	Yes	Yes	Yes	Yes	Yes	Yes	No	7
Tang et al. (Tissue Cell)	Yes	Yes	No	Yes	No	Yes	Yes	No	5
Zhang et al.	Yes	Yes	Yes	Yes	Yes	Yes	Yes	No	7
He et al.	Yes	Yes	Yes	Yes	Yes	Yes	No	No	6
Li et al.	No	Yes	Yes	Yes	Yes	Yes	Yes	No	6
Ma et al.	No	No	Yes	Yes	Yes	Yes	Yes	No	5
Wang et al.	Yes	Yes	Yes	Yes	Yes	Yes	Yes	Yes	8
Wasnik et al.	Yes	Yes	Yes	Yes	Yes	Yes	Yes	Yes	8
Xue et al.	Yes	Yes	Yes	Yes	Yes	Yes	Yes	No	7
He et al.	Yes	Yes	Yes	Yes	Yes	Yes	Yes	No	7
Jin et al.	Yes	Yes	Yes	Yes	Yes	Yes	Yes	No	7
Lin et al. (Tissue Eng Part A)	No	Yes	Yes	Yes	Yes	Yes	Yes	No	6
Nathan et al.	Yes	Yes	Yes	Yes	Yes	Yes	Yes	Yes	8
Sadowska et al.	Yes	Yes	Yes	Yes	Yes	Yes	Yes	No	7
Tang et al. (J Tissue Eng Regen Med)	Yes	Yes	Yes	Yes	Yes	Yes	Yes	Yes	8
Wang et al.	Yes	Yes	Yes	Yes	Yes	Yes	Yes	No	7
Wei et al.	Yes	Yes	Yes	Yes	Yes	Yes	Yes	No	7
Wendler et al.	Yes	Yes	Yes	Yes	Yes	Yes	Yes	No	7
Wu et al.	Yes	Yes	Yes	Yes	Yes	Yes	Yes	Yes	8
Yang et al.	Yes	Yes	No	Yes	Yes	Yes	Yes	No	6
Zhu et al.	Yes	No	No	Yes	Yes	Yes	Yes	Yes	6
Gao et al.	Yes	Yes	Yes	Yes	Yes	Yes	Yes	No	7

## Appendix

### A. Mesh Terms and Free Words

Mesh terms:

1. Mesenchymal Stem Cells
2. Macrophages
3. Bone regeneration

Free words:

1. Stem Cell, Mesenchymal
2. Stem Cells, Mesenchymal
3. Mesenchymal Stem Cell
4. Bone Marrow Mesenchymal Stem Cells
5. Bone Marrow Stromal Cells
6. Bone Marrow Stromal Cell
7. Bone Marrow Stromal Cells, Multipotent
8. Multipotent Bone Marrow Stromal Cells
9. Adipose-Derived Mesenchymal Stem Cells
10. Adipose Derived Mesenchymal Stem Cells
11. Mesenchymal Stem Cells, Adipose-Derived
12. Mesenchymal Stem Cells, Adipose Derived
13. Adipose-Derived Mesenchymal Stromal Cells
14. Adipose Derived Mesenchymal Stromal Cells
15. Adipose Tissue-Derived Mesenchymal Stem Cells
16. Adipose Tissue Derived Mesenchymal Stem Cells
17. Adipose Tissue-Derived Mesenchymal Stromal Cells
18. Adipose Tissue Derived Mesenchymal Stromal Cells
19. Mesenchymal Stromal Cells
20. Mesenchymal Stromal Cell
21. Stromal Cell, Mesenchymal
22. Stromal Cells, Mesenchymal
23. Multipotent Mesenchymal Stromal Cells
24. Mesenchymal Stromal Cells, Multipotent
25. Mesenchymal Progenitor Cell
26. Mesenchymal Progenitor Cells
27. Progenitor Cell, Mesenchymal
28. Progenitor Cells, Mesenchymal
29. Wharton Jelly Cells
30. Wharton's Jelly Cells
31. Wharton's Jelly Cell
32. Whartons Jelly Cells
33. Bone Marrow Stromal Stem Cells
34. Bone Marrow-Derived Macrophages
35. Bone Marrow Derived Macrophages
36. Bone Marrow-Derived Macrophage
37. Macrophage, Bone Marrow-Derived
38. Macrophages, Bone Marrow-Derived
39. Monocyte-Derived Macrophages
40. Monocyte Derived Macrophages
41. Macrophage
42. Macrophages, Monocyte-Derived
43. Macrophage, Monocyte-Derived
44. Macrophages, Monocyte Derived
45. Monocyte-Derived Macrophage
46. Bone Regenerations
47. Regeneration, Bone
48. Regenerations, Bone
49. Osteoconduction

### B. Recent Queries in PubMed: Search, Query, and Items Found

Please find Figure 3 below for the searching record in PubMed.

### C. Embase: Session Results

Please refer to Figure 4 below for the searching record in Embase.

### D. Methodological Quality Assessment Document (the Number of “Yes” Answers Was Counted for Each Study to Give a Total Score out of 8)

Please find Tables 4 and 5 below for the quality criteria which are specific to different paragraphs.

### F. Characteristics of Selected Studies

The induction methods of macrophage phenotypes can be roughly divided into 3 categories: (1) induction by biomaterials, (2) induction by cytokine combination, and (3) induction with gene-modified cells. Refer to the induction by cytokine combination, IFN-*γ* and LPS were most commonly for M1 induction, and IL-4 was for M2 induction. Flow cytometry analysis and real-time PCR were the most common assessments to pinpoint the subtypes of macrophages. CD11C, CCR7, TNF-*α*, and CD86 were used to identify M1 macrophages, and CD206, CD36, and CD163 were used to recognize M2 identification in flow cytometry analysis within selected papers. Regarding M1 and M2 marker genes, TNF-*α*, IL-6, IL-1*β*, IFN-*γ*, iNOS, CD86, and OSM represented M1 macrophages, while Arg1, CD206, CD163, IL-10, and Mrc1 were used for M2 macrophages. With respect to measuring the maturation of MSC osteogenesis, osteoblast-related genes, such as *ALP*, *OCN*, *OPN*, *COLI*, *RUNX2*, *IBSP*, and *BMP2*, were detected by real-time PCR and Western blot, and secreted proteins, such as *BMP*, *OSM*, *OPG*, *sRANKL*, and *MCSF*, were detected in ELISA. Alizarin Red S staining was used to evaluate calcium deposition/mineralization status during MSC osteogenesis.
